# Raman Spectroscopic Differentiation of *Streptococcus pneumoniae* From Other Streptococci Using Laboratory Strains and Clinical Isolates

**DOI:** 10.3389/fcimb.2022.930011

**Published:** 2022-07-22

**Authors:** Marcel Dahms, Simone Eiserloh, Jürgen Rödel, Oliwia Makarewicz, Thomas Bocklitz, Jürgen Popp, Ute Neugebauer

**Affiliations:** ^1^ Leibniz Institute of Photonic Technology Jena (a member of Leibniz Health Technologies), Jena, Germany; ^2^ Institute of Physical Chemistry and Abbe Center of Photonics, Friedrich Schiller University, Jena, Germany; ^3^ Center for Sepsis Control and Care, Jena University Hospital, Jena, Germany; ^4^ Institute for Medical Microbiology, Jena University Hospital, Jena, Germany; ^5^ Institute of Infectious Diseases and Infection Control, Jena University Hospital, Jena, Germany

**Keywords:** pneumococcus, bacteria, raman spectroscopy, binary PLS-DA classification models, streptococcus, clinical isolates, chemometrics

## Abstract

*Streptococcus pneumoniae*, commonly referred to as pneumococci, can cause severe and invasive infections, which are major causes of communicable disease morbidity and mortality in Europe and globally. The differentiation of *S. pneumoniae* from other *Streptococcus* species, especially from other oral streptococci, has proved to be particularly difficult and tedious. In this work, we evaluate if Raman spectroscopy holds potential for a reliable differentiation of *S. pneumoniae* from other streptococci. Raman spectra of eight different *S. pneumoniae* strains and four other *Streptococcus* species (*S. sanguinis*, *S. thermophilus*, *S. dysgalactiae*, *S. pyogenes*) were recorded and their spectral features analyzed. Together with Raman spectra of 59 *Streptococcus* patient isolates, they were used to train and optimize binary classification models (PLS-DA). The effect of normalization on the model accuracy was compared, as one example for optimization potential for future modelling. Optimized models were used to identify *S. pneumoniae* from other streptococci in an independent, previously unknown data set of 28 patient isolates. For this small data set balanced accuracy of around 70% could be achieved. Improvement of the classification rate is expected with optimized model parameters and algorithms as well as with a larger spectral data base for training.

## Introduction

*Streptococcus pneumoniae*, commonly referred to as pneumococci, are Gram-positive bacteria that can cause severe local and invasive infections, such as pneumonia, sinusitis, rhinitis, otitis media, mastoiditis, meningitis and bacteremia (World Health Organization, 2022; Weiser et al., 2018; Brooks and Mias, 2018; Loughran et al., 2019; Hall et al., 2021). The bacteria are transmitted by droplet infection and settle on the mucosa of the upper respiratory tract. Pneumococcal infections are major causes of morbidity and mortality from communicable disease worldwide. Up to ten percent of adults and up to 65% of children are asymptomatically colonized by pneumococci (Song et al., 2013; Hall et al., 2021). However, in the presence of weakened immune defenses, e.g., due to immune senescence or immunosuppressive therapy, they can easily spread and cause severe disease progression (Brooks and Mias, 2018; Gierke et al., 2021).

Due to the rapid need for action in pneumonia, antibiotic therapy is often initiated in advance of pathogen identification, after which microbiological diagnosis, including cultivation of the pathogen, is almost impossible (Welte, 2016). If the time-consuming cultivation is successful, a further differentiation of *S. pneumoniae* from other oral streptococci is sometimes difficult as they share similar phenotypic characteristics on blood agar plates, namely alpha-hemolysis resulting in a greenish agar surrounding the bacterial colony. Due to the greenish color on blood agar plates, those non-pneumococcal streptococci are called viridans streptococci (**Supplemental Figure S1**). In classical microbiology, the optochin test is used to differentiate *S. pneumoniae* from viridans streptococci.

As for life-threatening bacterial pneumonia and especially invasive infection the diagnostic result is needed urgently, various cultivation-independent methods have been developed in recent years to detect *S. pneumoniae* (Varghese et al., 2017). These include molecular biological detection methods of pneumococcal antigens using polymerase chain reaction (PCR) and rapid tests detecting pneumococcal antigens directly from patient material like urine and sputum without prior enrichment (Sadowy and Hryniewicz, 2020). Hereby, the urine-based test can provide a first rapid indication of a possible pneumococcal infection. However, it shows lower sensitivity in some cases leading to incorrect diagnoses (Song et al., 2013; Fukushima et al., 2015). Detection methods using polymerase chain reaction (PCR) are faster than cultivation-based methods, and the genes used for detection can be found in other, closely related streptococci species as well, which limits the application in a routine microbiological setting and diagnostics, especially in severe ventilation-associated pneumonia (Song et al., 2013; Torres et al., 2016). Matrix-Assisted Laser Desorption-Ionization Time-Of-Flight Mass Spectrometry (MALDI-TOF MS) with the continuously improving databases is advancing to a valuable tool for microbial species identification after a cultivation step (Hou et al., 2019), despite initial limitations in the correct differentiation of *S. pneumoniae* from other species in the *Streptococcus mitis* group (Marín et al., 2017).

In the last years, light-based methods, like Raman spectroscopy, gained interest for rapid diagnostics (Wang et al., 2021; Lee et al., 2021). Studies have already demonstrated successful differentiation of *S. pneumoniae* from other Gram-positive and Gram-negative bacteria, based on the different molecular composition of each bacterial species (Kloß et al., 2015; Kloß et al., 2015; Ayala et al., 2017). Enrichment of bacteria in a cultivation step is theoretically not necessary, as a single bacterium is sufficient for identification (Strola et al., 2014; Novelli-Rousseau et al., 2018).

The aim of this study is to estimate the potential of Raman spectroscopy for differentiation of *S. pneumoniae* from other streptococci as proof-of-concept study. Therefore, we first studied the spectral characteristics of cultivated laboratory strains which comprised eight different *S. pneumoniae* strains (seven different serotypes) as well as four other *Streptococcus* species colonizing the upper respiratory tract, such as *S. dysgalactiae* as well as *S. pyogenes*, *S. thermophilus* and *S. sanguinis*, which show alpha-hemolysis when grown on blood agar plates similar to *S. pneumoniae*. The spectral data base was extended with 59 patient isolates (31 *S. pneumoniae* and 28 other viridans streptococci) for training of different classification models. These were used to predict the identity (*S. pneumoniae* vs. other streptococci) of further 28 unknown clinical isolates and compared to reveal future potential of optimization and raise awareness for potential pitfalls of modelling used for medical applications. While Raman spectroscopic bacterial identification is possible with single bacteria, we decided to work with cultivated bacteria in this proof-of-principle study where we address the general question if a Raman-based differentiation between *S. pneumonia* and closely related other streptococci is possible. Thus, at this point of the study, we still exclude special, cultivation-independent sample preparation strategies.

## Materials and Methods

### Laboratory Strains

Laboratory strains and patient isolates from the culture collection of the Institute of Infectious Diseases and Infection Control as well as of the culture collection of the Medical Microbiology of the Jena University Hospital were used in this study.

To ensure capturing a naturally occurring variance of the pneumococci, eight characterized laboratory strains of *S. pneumoniae* representing seven different serotypes (1, 3, 6B, 14, 15B, 19A, 19F) were used (**Supplementary Table S1**). Selection of serotypes was based on prevalence in community acquired pneumonia and also epidemiological data from the Germany National Reference Center for Pneumococci (Rose et al., 2021). Due to their prevalence, those serotypes are also included in the pneumococcal vaccines Pneumovax and Prevenar (here 15B is missing). Some of the selected serotypes (6B, 14, 19A and 19F) are also known to be related to drug-resistant isolates and Serotype 3 shows some specific characteristics in morphology (smallest capsid-units, but bigger capsule and bigger colonies on the plate) and more severe disease progression, most likely due to its capsid properties (Weber et al., 2012).

Additional laboratory *Streptococcus* strains of four representative species commonly colonizing the mouth, nose and throat of humans were chosen: *S. dysgalactiae* and *S. pyogenes, S. thermophilus* and *S. sanguinis* (**Supplementary Table S1**).

Laboratory strains were taken from cryoculture (-80°C) and cultivated on blood agar plates (BD Columbia Agar with 5% sheep blood, Becton Dickinson, Germany) at 37°C for 20 to 24 hours. Six bacterial strains were additionally cultivated for an extended period of three days (**Supplemental Table S1**), to capture growth phase heterogeneity in the data set. Bacteria from all used strains were cultivated and measured at least on three different days, each day representing a different replicate.

### Patient Isolates

Clinical isolates were collected and characterized by the Institute of Medical Microbiology, Jena University Hospital during routine bacteriological examinations between March and April 2017.

In total, 87 streptococci (*S. pneumoniae* or other viridians streptococci) were isolated from various patient materials such as blood, bronchoalveolar lavage, sputum, tracheal and bronchial secretions as well as from swabs of the oropharynx, eye and wound (**Supplemental Table S1B**). After cultivation on blood agar plates (Columbia Agar + SB Plus, Oxoid™, Germany) at 37°C (+ 5% CO_2_) overnight, identification of *S. pneumoniae* was performed in routine microbiological diagnostics and included optochin (ethylhydrocupreine hydrochloride) test (ThermoFisher, Wesel, Germany), genetic and visual assessment, as well as MALDI-TOF-MS (Vitek MS, bioMerieux, Nürtingen, Germany) identification. Serotyping of *S. pneumoniae* was performed by the National Reference Center for Streptococci (Aachen, Germany) in cases of invasive diseases. Five different serotypes were found (11A, 15C, 16F, 22F, 23A). Other viridans streptococci were identified by hemolysis on blood agar plates together with optochin testing to exclude pneumococci, but no further determination of species took place. Agar plates were stored for an extended period (~4 days) at 4°C until further use for Raman spectroscopic analysis. After visual inspection, small colonies, but also overgrown and older colonies were re-cultivated for 24 to 48 hours at 37°C prior to spectral characterization.

Identification results of the first 59 patient isolates were made available for training the classification models, while the identification of the last 28 patient isolates was kept disclosed until Raman prediction was finished.

### Sample Preparation for Raman Spectroscopic Measurement

Bacteria from the agar plates were harvested from colonies, resuspended in PBS (Dulbecco, Biochrome GmbH, Germany) and then centrifuged. This washing step was repeated once with aqua bidest by centrifugation at 7000 g for 10 min. The purpose of this washing step was to remove any possible contamination with agar that could have been accidentally collected from the plate and would influence the Raman signal. The bacterial pellet was resuspended in 15 to 100 μl aqua bidest. Three to five μl of the bacterial suspension was applied to a calcium fluoride slide (Crystal GmbH, Germany) and allowed to dry. The dripping and drying was repeated two more times, resulting in a dense bacterial coating.

### Raman Spectroscopic Measurements

A commercial Raman micro-spectrometer (CRM alpha 300, WITec GmbH, Germany) coupled to a frequency doubled Nd:YAG laser (532 nm, 15 mW in sample plane) was used. A 100✕/NA 0.75 microscope objective (Carl Zeiss GmbH, Germany) was used to focus the laser onto the sample and to record the scattered light. Scattered light (Stokes) was collected with an optical fiber (100 μm diameter) and separated with a 600 grooves/mm grating (blaze 500 nm) and detected with a CCD camera (DU401A BV-532, ANDOR, 1024✕127 pixels, cooled to -60°C).

On each dried bacterial droplet, a series of 50 individual spectra with acquisition time of 1s was recorded at ten different positions, so that 500 spectra were recorded per sample. To reduce fluorescence background in some samples, fluorescence was bleached with extended laser illumination prior to spectrum acquisition. Raman spectra of silicon and 4-acetamidophenol (Sigma Aldrich, Germany) were recorded on a daily basis prior to start of sample measurements as reference samples and for calibration of the system.

### Data Analysis

Data analysis was performed using in-house written scripts in the R programming language (version 3.6.3) (Team RC, 2020). Handling of spectral data was performed using the packages hyperSpec (Beleites and Sergo, [v 0.100.0]) and dplyr (Wickham et al., 2022). Plots are created using the packages graphics (Team RC, 2020) and ggplot2 (Wickham, 2016).

Spectral preprocessing involved the following steps. First, cosmic spikes were removed by replacing the data points of the spikes with adjacent data points using neighboring data points. Wavenumber calibration was conducted on daily basis by acquisition of 50 spectra of 4-acetamidophenol. These were averaged and a third order polynomial was fitted to 17 band positions between 465 and 3327 cm^-1^ to shift the wavenumber axis accordingly. Raman band positions in the spectra were determined within a range of 5 data points. Spectra were interpolated onto a wavelength axis from 400 to 3800 cm^-1^ with a data point distance of 4 cm^-1^. Background correction was performed with asymmetrical least squares from the baseline package (lambda = 4.5, p = 0.01) (Liland et al., 2010). Afterwards, spectral outliers (e.g. burned or highly fluorescent spectra) were removed by means of Euclidean distance, where spectra were discarded, which exceeded the average Euclidean distance plus 2.5 times its standard deviation of the lowest 5% Euclidean distances. Finally, ten individual spectra of each 50-spectra-series were aggregated into a single spectrum by averaging. Aggregated spectra were truncated to the spectral region from 500-1750 cm^-1^ and 2800-3050 cm^-1^. Vector normalization of aggregated spectra was used in one model while it was omitted in the other.

The data set was split into a training and test data set according to measurement time (**Table 1**). Binary classification models using partial least square regression discriminant analysis (PLS-DA) (own implementation based on pls package (Liland et al., 2021)) were built to differentiate *S. pneumoniae* and other streptococci. Models were trained on spectra with and without vector normalization, respectively. For optimization of hyperparameters (number of used components in PLS regression and discrimination threshold for discriminant analysis), a 20-times 10-fold cross validation was performed. Folds were built ensuring complete isolates belonging to a single fold and stratified in isolates between *S. pneumoniae* and other streptococci. Nevertheless, this leads to slightly different ratios between both classes, as the overall number of aggregated spectra is slightly different between isolates. The best model was selected to be nearest (Euclidean distance) to the optimal model performance (100% true-positive rate and 0% false-positive rate) in using receiver operating characteristic (ROC) curves (Sing et al., 2005) over discrimination threshold. To avoid model overfitting, hyperparameter optimization was achieved by averaging the obtained distances of all 20 iterations and choosing the lowest component number, which equals or is lower than the lowest average distance plus corresponding standard deviation [known as one standard error rule (Hastie et al., 2009)]. For the so chosen component number, threshold optimization using all 20 iterations was repeated.

**Table 1:** Overview of number of aggregated spectra used for modelling and testing.

Type	Training spectra (laboratory strains + patient isolates)	Test*: spectra (patient isolates)
*S. pneumoniae*	1177 (8) + 1142 (25) = 2319 (33)	122 (3)
Other streptococci	682 (4) + 1575 (34) = 2257 (38)	1157 (25)
total	1859 (12) + 2717 (59) = 4576 (71)	1279 (28)

The number in brackets gives the number of independent laboratory strains/patient isolates. (more detailed information about laboratory strains and patient isolates is given in **Supplementary Table S1**).

*Identification of strains in the test data set was not disclosed until Raman prediction was finished.

## Results and Discussion

### Spectroscopic Features of the Laboratory Strains

**Figure 1:**
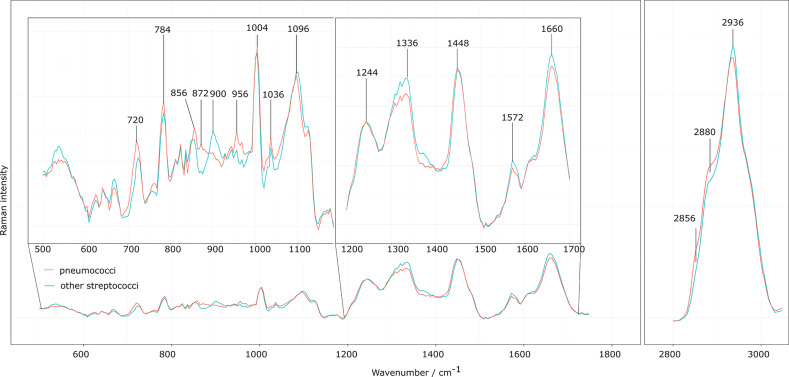
Raman mean spectra of the laboratory strains. Preprocessed Raman mean spectra of the eight different *S. pneumoniae* strains (orange) and the four different non-pneumococcal *Streptococcus* species (turquois) presented as overlaid spectra to visualize spectral differences. The insets highlight Raman bands of interest discussed in the corresponding assignment in **Table 2**.

**Figure 2:**
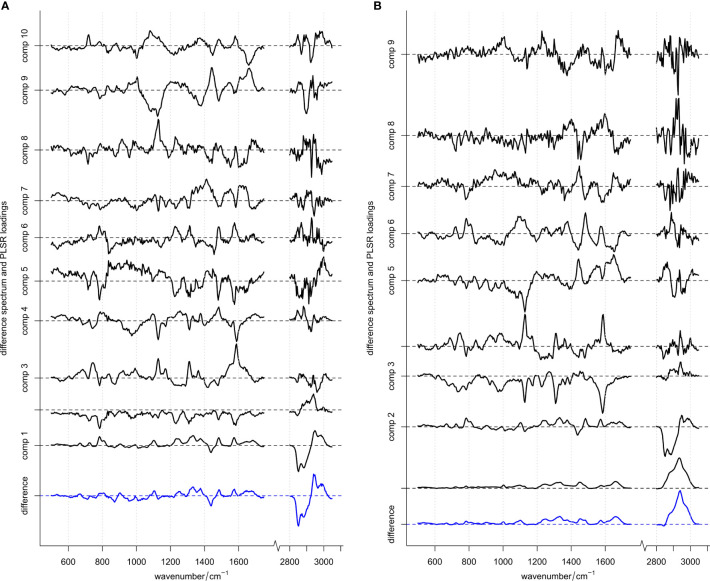
Difference spectrum (blue, bottom) and PLSR loadings (black) for models. **(A)** With and **(B)** without vector normalization applied to the spectral data in the training set. Difference spectra (computed by subtracting the mean spectrum of *S. pneumoniae* from the mean spectrum of other streptococci) are scaled appropriately matching the loadings scale and are depicted in blue. Loadings are shown in black with increasing components organized from bottom to top. For each spectrum the “zero line” on the y-axis (contribution of Raman intensity) is indicated with a dotted line. ROC curve using these 10 or 9 loadings, respectively, are depicted in **Supplementary Figure S4**.

Mean spectra of the eight laboratory strains of *S. pneumoniae* and the four other *Streptococcus* strains are found in **Supplemental Figure S2**. The eight different pneumococcal strains were selected to represent seven different serotypes (1, 3, 6B, 14, 15B, 19A, 19F; **Supplementary Table S1**), so that the variation of the pneumococcal polysaccharide capsules is included in the training data set. However, as the aim of this manuscript is to differentiate *S. pneumoniae* from other streptococci, we considered in the following the mean spectrum of the different *S. pneumoniae* strains and compared it to the mean spectrum of the other four selected streptococci, as displayed in the overlay in **Figure 1**. Minor spectral differences between *S. pneumoniae* and other streptococci are visible and also found in the computed difference spectrum in **Figure 2** (left side, bottom). The comparison is summarized in the last two columns of **Table 2**.

**Table 2:** Typical Raman bands found in streptococci spectra (**Figure 1**), assignment to functional groups and rough estimation of relative intensity of the respective Raman bands in *S. pneumoniae* (*S. p.*) and other streptococci (o. *S*.).

Raman	Characteristic Raman bands and assignment (Maquelin et al., 2002; Neugebauer et al., 2010; Krafft et al., 2012; Rygula et al., 2013; Czamara et al., 2015; Wiercigroch et al., 2017)					
ν [cm^-1^]	DNA/RNA	Proteins	Lipids	Carbohydrates	*S. p*.	o. *S*.
2936	**	CH_3 (str)_, CH_2 (str)_	CH_3 (str)_	CH_2 (str)_, CH _(str)_	+	++
2880		CH_2 (str)_	CH_2 (str)_		++	+
2856		CH_2 (str)_	CH_2 (str)_		++	+
1660		Amid I _(C=O)_	C=C _(str)_		+	++
1572	G/A _(Ring, str)_				+	+ +
1448		C-H _(def)_	CH_2_/CH_3 (def)_		+	+
1336-1376	T/A/G	C-H _(def)_, Trp		CH, CH_2 (def)_	+	+++
1244		Amid III _(C-N, N-H)_			+	+
1096			C-C	COC glycosidic bond	++	+
1004		Phe			+	+
956		Trp, Val, Tyr		COC glycosidic bond	++	+
872		Trp	N^+^(CH_3_)_3_ C-O-O _(skeletal)_		++	
856		Tyr, Pro		CC, COC, glycosidic bond	++	+
784	C/U/T _(str)_				+ +	+
720	A		N^+^(CH_3_)_3 (str)_		+ +	+

**No exact information possible, S. p., S. pneumoniae; o. S., other streptococci; str, stretching vibration; def, deformation vibration; A, adenine; G, guanine; C, cytosine; T, thymine; U, uracil; Phe, phenylalanine; Trp, tryptophan; Tyr, tyrosine; Val, valine; Pro, proline; +, present; ++/+++, increased.

A slightly higher Raman band 720 cm^-1^ is found for *S. pneumoniae*. This band is generally assigned to adenine, but also to the N^+^(CH_3_)_3_ head group of lipids and choline. Another N^+^(CH_3_)_3_ Raman band around 872 cm^-1^ (Neugebauer et al., 2010; Czamara et al., 2015) is also found with increased relative intensities in *S. pneumoniae*. The choline incorporated into the pneumococcal cell wall might be responsible for these spectral differences, as it is only found in a few prokaryotes, such as *S. pneumoniae*, or also *Haemophilus influenzae* (Fan et al., 2003; Schneewind and Missiakas, 2014).

*S. pneumoniae* has a polysaccharide capsule with the exact composition and thickness varying between the serotypes. The capsule can consist of two to eight different saccharides in different order and linkages and with varying substitution patterns that include O-acetyl, phosphoglycerol and pyruvyl acetal (Geno et al., 2015). A higher Raman intensity was observed at 856 cm^-1^ for *S. pneumoniae*, possibly due to COC stretching vibrations and CC deformation vibrations in glycosidic compounds, but also attributed to amino acids tyrosine and proline (Maquelin et al., 2002; Rygula et al., 2013; Wiercigroch et al., 2017). Furthermore, a higher Raman intensity was observed for *S. pneumoniae* around 956 cm^-1^, which is also attributed to COC stretching vibrations (α-D-1,4-glycosidic bond, α-D-1,6-glycosidic bond) in polysaccharides as well as the amino acids tryptophan and valine (Rygula et al., 2013; Wiercigroch et al., 2017). Consistent with the observations of Kloß *et al.*, a higher intensity in the 2856 cm^-1^ and 2880 cm^-1^ range was observed for *S. pneumoniae* (Kloß et al., 2015). These bands are mainly assigned to CH_2_ stretching vibrations in lipids. A lower Raman intensity for *S. pneumoniae* was observed around 2936 cm^-1^ of the CH_3_ stretching vibrations, which are mainly found in proteins. It can be seen that *S. pneumoniae* has a different lipid-protein ratio than other oral streptococci.

*S. pneumoniae* has a lower relative Raman intensity in the range of 1336 cm^-1^ to 1376 cm^-1^ compared to other streptococci. This region is assigned to the nucleic acids (adenine, thymine and guanine) and the amino acid tryptophan as well as the CH deformation vibrations in proteins and polysaccharides (Neugebauer et al., 2010; Rygula et al., 2013; Czamara et al., 2015; Wiercigroch et al., 2017). Other small differences ran through the entire lower spectral range. They could mainly serve to differentiate *S. pneumoniae* from individual *Streptococcus* species.

All in all, *S. pneumoniae* shows clear spectral differences in the observed spectral range. Nevertheless, similar heterogeneities are also found for other *Streptococcus* species making that group rather inhomogeneous in terms of later classification into a single group.

### Training and Optimization of Classification Model

Binary classification models based on partial least squares regression (PLSR) combined with discriminant analysis (DA) were automatically optimized with a training data set comprising laboratory strains and patient isolates (**Table 1**) to utilize the small Raman spectroscopic differences between *S. pneumoniae* and other streptococci (**Figure 1** and **Table 2**) for automated differentiation. A commonly used spectral pre-treatment involves vector normalization (Lee et al., 2021) to account for variations in spectra due to sample differences (e.g., sample thickness) or measurement differences (e.g., focus variations). However, as vector normalization leads to projection of the data onto the surface of a multi-dimensional sphere, it might introduce some non-linear effects and thus, might badly affect the regression step in the PLS-DA model. In order to investigate this effect, we have used the aggregated spectra either with or without vector normalization.

Automated ROC curve optimization (**Supplementary Figures S3** and **S4**) suggested to use 10 or 9 PLSR components for both optimized models, respectively (i.e., one model with normalized data, one model without vector normalization). **Figure 2** depicts those components together with the computed difference spectrum between the two classes *S. pneumoniae* and other streptococci. In both models, the respective difference spectrum is very similar to the first component, indicating that the underlying spectral difference is captured by the model. Due to missing intensity normalization (as vector normalization was omitted), component 1 of the model without vector normalization is dominated by an offset spectrum representing an absolute difference spectrum. However, component 2 of this model (**Figure 2B**) is very similar to component 1 of the model using normalized data (**Figure 2A**). Also, for higher components, spectral features are very similar in both models (despite being flipped around 0) and display spectral features discussed above and summarized in **Table 2**. This indicates that the models rely on real spectral information already confirmed and interpreted by bio-spectroscopic knowledge. The PLS regression coefficients (**Supplementary Figure S5**) summarize relevant spectral features used by the models. Those features are found with almost equal peak heights across the whole spectrum, indicating that spectral information is used across the whole spectrum with similar importance.

**Figure 3:**
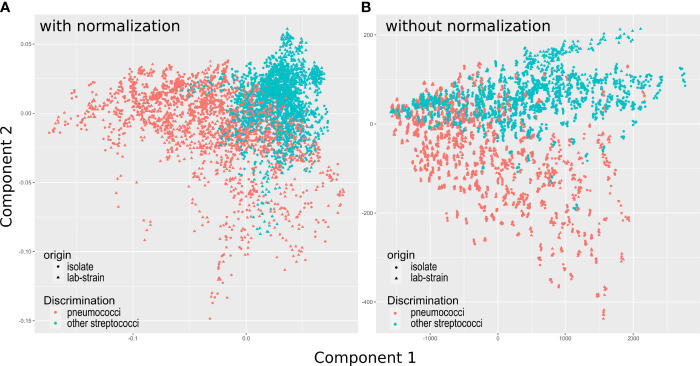
Score plots for components 1 and 2 for PLS models. **(A)** Shows scores for the model using vector normalization, **(B)** The model using no normalization. Pairs plot of all 7 PLSR scores are depicted in **Supplementary Figure S5** and with different color coding in **Supplementary Figure S6**. Model performance during auto-prediction (using the training data set) and during prediction of unknown test data set is shown in **Table 3**.

A visualization of the discrimination of *S. pneumoniae* and other streptococci with both models during regression phase is depicted in the 2-dimensional PLS score in **Figure 3** (a visualization with higher components is shown in **Supplementary Figures S6, S7**). Spectra of the different *S. pneumoniae* strains show a high variation in score space. Laboratory strains and patient isolates from the training data set are equally distributed in the score space, indicating principal comparability of laboratory strains and isolates. The broad spectral variations might arise from chemical differences between strains and serotypes in the very inhomogeneous class of *S. pneumoniae*. Large variation in the chemical structure of the polysaccharide capsule in the different serotypes have been described (Bentley et al., 2006).

In the group of other streptococci all non-*S. pneumoniae* streptococci are included. For the laboratory strains, this comprises four different species. For the clinical isolates no identification on species level was available during this study, however, it is known that all patient isolates are viridans streptococci, i.e., streptococci that are alpha-hemolytic and produce a green coloration on blood agar plates (see also **Supplemental Figures S1A–D**). Also for this group, a high heterogeneity is expected and found in **Figure 3**.

**Table 3** summarizes the auto-prediction performance of the two different models, i.e., the model with and without normalization of the spectral data. The performance is higher for the model using vector normalization. As optimization is performed during cross-validation and thus subject to randomness of splitting, the stability of the optimization procedure was investigated by using another outer 5-times 5-fold cross-validation. Within this cross-validation, four folds were used for automated model optimization as already described above and the remaining fold was used for determining the model performance. Note, models might suffer from a reduced data set available for model optimization as only 4/5 of the data set is available compared to the model optimization used for the final models. Results of this investigation are visualized in **Supplemental Figure S8**: For both types of pre-processing, a broad range of hyperparameters is seen during cross-validation. Using no vector normalization appears to be slightly more stable, especially concerning selection of number of components and discrimination threshold. The balanced accuracy is ranging from <80% to >95% and <80% to 95% for models with and without vector normalization, respectively. A similar broad variance can be seen for sensitivity, which ranges from >95% to <60% for both preprocessing approaches. This broad range indicates that model optimization is currently not stable, thus parameters in the final models used above might not yet be optimal. This can also be seen in the corresponding regression coefficients showing clear differences over the course of 5 repetitions, being directly related to the number of chosen components during optimization (**Supplemental Figure S9**). A possible reason for the instability might be the slightly changing prevalence across different splittings during cross-validation in the relatively small data set. It is expected that model optimization becomes more stable with more patients/strains included into the model in future.

**Table 3:** Summary on model performance of the automatically optimized models during auto-prediction (training data set, left) and during prediction of unknown test data set (right).

	Auto-prediction (training data set)				Prediction of unknown test data			
Model	Sens.	Spec.	PPV	Bal. acc.	Sens.	Spec.	PPV	Bal. acc.
1 (with vector norm.)*	68.9 (78.8)	91.3 (89.5)	89.1 (86.7)	80.1 (84.1)	56.6 (33.3)	75.5 (72.0)	19.6 (12.5)	66.0 (52.7)
2 (without norm.)**	57.9 (51.5)	69.2 (76.3)	65.9 (65.4)	63.5 (63.9)	63.1 (66.7)	76.1 (76.0)	21.8 (25.0)	69.6 (71.3)

Numbers in brackets represent the corresponding value after majority vote per patient/isolate. A detailed overview of the prediction is given in **Supplementary Table S2A** (auto-prediction) and **S2B** (test data).

*Model 1: with vector normalization using 10 components, discrimination threshold: -0.01;

**Model 2: without normalization using 9 components, discrimination threshold: -0.091.

Sens … Sensitivity, Spec … specificity, PPV … positive predictive value, Bal. acc … balanced accuracy.

The positive class is *S. pneumoniae*.

### Validation of the Classification Model by Predicting Unknown Patient Isolates

Both binary PLS-DA classification models were used to predict the presence of *S. pneumoniae* in patients’ isolates in an unknown data set comprising 28 patient isolates (1279 aggregated Raman spectra recorded after the acquisition of the training data set). Additionally, to the prediction on individual spectra, a summarized prediction was performed by using a majority vote per patient based on predictions on individual aggregated spectra. **Table 3** summarizes the performances. A detailed patient-wise assignment is given in **Supplemental Table S2B**.

For the prediction of a small unknown data set balanced accuracies of around 70% with sensitivities and specificities ranging from 56% to 63% and 75% to 76% were obtained, respectively. At current state, this would not yet meet clinical performance criteria.

However, it needs to be emphasized, that the test set is very small with only 28 individual isolates, thus point estimates are very uncertain. Taking this into account, both models show similar overall performance. For an expected balanced accuracy of 70% based on 28 observations, the 95% confidence interval ranges from 54.2% to 86.3%. If we would have the same observed accuracy derived from 100 observations, the 95% confidence interval would shrink to 60.8% to 78.6%. This shows very impressively that further data is needed to reveal the real performance. A more generalized investigation on the link between sample size and performance point estimates is given by Beleites et al. (2013).

### Discussion of Statistical Data Analysis and Importance of Results

The unknown data set used for model validation contained only three cases of *S. pneumoniae*. This imbalanced test data set does not reflect the abundance ratio of *S. pneumoniae* and other streptococci used during model training. Using majority votes per patient to increase prediction robustness only worked out for models using no normalization, whereas the performance dropped remarkably for models using vector normalization.

It has to be noted that first attempts using MALDI-TOF mass spectrometry-based differentiation with small data sets resulted in similar prediction accuracies. Exemplarily, one study should be mentioned that reports a drastically improved differentiation accuracy when the database was increased from 4613 strains to 5627 strains: the misclassification of *S. mitis* species group isolates could be reduced from 66 to one out of 101 isolates (Harju et al., 2017). Our data base in the current proof-of-principle study contains only 71 laboratory strains/patient isolates and our test data set only 28 patient isolates. Thus, we believe that despite the shortcomings in the currently achieved prediction accuracy, our proof-of-concept study reveals the potential of the label-free Raman method. This could be demonstrated during the discussion of the spectral features which clearly highlight certain properties of the bacteria. However, it has to be noted that both classes that should be differentiated here with a binary classification model, i. e., *S. pneumoniae* and other streptococci, are very heterogeneous (from *S. pneumoniae* 92 different serotypes are described (Yother, 2011)), with high similarity also between strains of both classes. Thus, a larger data set is needed to build reliable classification models as can be estimated using sample size planning (Beleites et al., 2013) and was already experimentally proven using MALDI-TOF MS data (Harju et al., 2017).

In this contribution, a single data analysis algorithm, namely PLS-DA, was utilized. However, many other model algorithms and types have been reported to be powerful for building classification models of spectral data (Gautam et al., 2015; Byrne et al., 2016). These are e.g., one-class-classifiers like SIMCA (Soft Independent Modelling by Class Analogy), logistic regression, or other linear or non-linear models like support vector machines (SVMs), to just name a few. All models have their certain characteristics and advantages as well as disadvantages. The choice of the most appropriate approach is highly connected to the final application scenario (e.g., inference by other strains than streptococci, required level of identification of viridans streptococci or capsule identification requirements, to just name a few). If only *S. pneumoniae* would be in focus of interest, one-class-classifiers would be most suitable as they only model the class of interest (i.e., only identifying *S. pneumoniae*) without stating a class for all other cases by answering the question “Is it *Streptococcus pneumoniae*” only with “yes” or “no”. However, a detailed discussion on different models is beyond the scope of the manuscript.

The current model was optimized using the nearest (Euclidean distance) to optimal model performance, i.e., 100% true-positive rate and 0% false-negative rate. However, other optimization criteria, such as highest sensitivity (i.e., not to miss any *S. pneumoniae*) or specificity (i.e., to exclude all other streptococci), could also be used and should be selected to optimal match clinical requirements.

The automated optimization revealed seven components for the final model. However, visual inspection (with the eyes of a spectroscopist) of the components (**Figure 2**) indicated that higher components (>5) already contained increased noise. This means, that there is a certain chance that the model already starts to overfit. To evaluate this option, two more models were built in which only 4 components were used. The resulting PLS coefficient for the model is much less noisy (**Supplemental Figure S5**), while the predictions with the 4 component models perform almost comparable with the 10-component model (**Supplemental Table S2, S3**). An increased data set could also help here for stabilizing the model optimization.

## Conclusions and Recommendations

Compared to other label-free methods established in bacterial differentiation (such as MALDI-TOF MS where data bases contain more than 5600 different streptococcal strains), the data set used in our proof-of-principle study is relatively small, comprising in total 5855 spectra from 99 streptococcal strains/clinical isolates. Despite the small data set, it could be shown that a discrimination of *S. pneumoniae* from other oral streptococci by means of Raman spectroscopy is in principle possible (despite not yet optimal accuracies). The spectral features used by the model could be supported with known biological characteristics of *S. pneumoniae* and other streptococci. Test performance was similar to performance obtained during cross-validation. Thus, label-free Raman spectroscopy offers a high potential which should be exploited in future studies with larger data sets.

In our study, pathogens could be classified in one to two hours using micro-Raman spectroscopy and statistical data analysis after a pre-cultivation step. However, Raman spectroscopy offers in general the possibility of characterizing single individual bacteria. This means that with the right sample pretreatment steps, time-consuming cultivation could eventually be omitted. Therefore, we conclude, the presented Raman-based method offers a high potential for the timely and correct identification of *S. pneumoniae* which should be exploited in further studies.

The relatively small data set of highly heterogeneous streptococcal groups leaves room for further improvements in future studies. It is highly recommended to include more other streptococci as well as more serotypes of *S. pneumoniae* in the training data set. Also, improving the methodology could result in better classification accuracy. Here, we recommend to further optimize data pre-processing to capture spectral effects occurring in the cultivation of patient’s isolates as well as apply and utilize more potentially sophisticated model algorithms (one-class-classifiers, other linear or non-linear algorithms) which can be applied if a larger data set is available. Attention should be paid to avoid overfitting and to improve optimization heuristics in order to obtain reliable results.

## Data Availability Statement

The raw data supporting the conclusions of this article will be made available by the authors, without undue reservation.

## Author Contributions

UN, JR, SE, MD designed the study, OM, JR collected and provided biological material, SE, JR performed measurements, MD analyzed data, MD analyzed data, TB reviewed data analysis, all authors discussed results, SE, UN, MD drafted the manuscript, all authors corrected and approved the final manuscript.

## Funding

Funding by the BMBF *via* the Centre for Sepsis Control and Care (FKZ 01EO1502), *via* the Forschungscampus InfectoGnostics (FKZ 13GW0096F) and *via* ReHwIN (13GW0432F), the Leibniz Association *via* the Leibniz Science Campus InfectoOptics (W8/2018), and the European Union *via* the EU Horizon 2020 Marie Skłodowska-Curie European Training Network IMAGE-IN (Grant agreement No. 861122) is highly acknowledged. Further, we gratefully acknowledge The CSCC Core Unit Biophotonics as part of the Jena Biophotonic, Imaging Laboratory (JBIL, FKZ PO633/29-1, BA 1601/10-1) and the ThIMEDOP (Thüringer Innovationszentrum für Medizintechnik-Lösungen, FKZ IZN 2018 0002) for providing the infrastructure. The work is supported by the BMBF funding program Photonics Research Germany (FKZ: Leibniz-IPHT LPI-BT4 13N15708 and FSU LPI-BT1 13N15466) and is integrated into the Leibniz Center for Photonics in Infection Research (LPI).

## Conflict of Interest

The authors declare that the research was conducted in the absence of any commercial or financial relationships that could be construed as a potential conflict of interest.

## Publisher’s Note

All claims expressed in this article are solely those of the authors and do not necessarily represent those of their affiliated organizations, or those of the publisher, the editors and the reviewers. Any product that may be evaluated in this article, or claim that may be made by its manufacturer, is not guaranteed or endorsed by the publisher.
