# Supplementary material for: Raman Spectroscopic Differentiation of Streptococcus pneumoniae From Other Streptococci Using Laboratory Strains and Clinical Isolates
Source: Front Cell Infect Microbiol. 2022 Jul 22;12:930011. doi: 10.3389/fcimb.2022.930011 (PMC9353136; doi:10.3389/fcimb.2022.930011)
Supplement: Supplementary file 1 [file DataSheet_1.pdf]

Supplemental Material for

Differentiation of *Streptococcus pneumoniae* from other streptococci from patient isolates using Raman spectroscopy

Marcel Dahms[1,2#], Simone Eiserloh[1,3#], Jürgen Rödel[4], Oliwia Makarewicz[3,5], Thomas Bocklitz[1,2], Jürgen Popp[1,2], Ute Neugebauer[1,2,3] *

[1] *Leibniz Institute of Photonic Technology Jena (a member of Leibniz Health Technologies)*
[2] *Institute of Physical Chemistry and Abbe Center of Photonics, Friedrich Schiller University*
[3] *Center for Sepsis Control and Care, Jena University Hospital*
[4] *Institute for Medical Microbiology, Jena University Hospital*
[5] *Institute of Infectious Diseases and Infection Control, Jena University Hospital*

* *Email: ute.neugebauer@leibniz-ipht.de, Phone: +493641206103*
[#] *shared first authorship*

Table of content:

Supplemental Figure S1: Macroscopic characteristics of some *Streptococcus* species on blood agar plates S2

Supplemental Figure S2: Mean spectra of laboratory strains S3

Supplemental Figure S3: ROC curve for optimization of PLS-DA model based on vector normalized data S4

Supplemental Figure S4: ROC curve for optimization of PLS-DA model based on data without vector normalization S5

Supplemental Figure S5: PLS coefficients for all four models S6

Supplemental Figure S6: Binary PLSR model for the differentiation of *S. pneumoniae* and other streptococci (Pairs plot of PLS scores colored according to classes) S7

Supplemental Figure S7: Binary PLSR model for the differentiation of *S. pneumoniae* and other streptococci (Pairs plot of PLS scores colored according to species) S8

Supplemental Figure S8: Model stability during outer cross-validation. S9

Supplemental Figure S9: Regression coefficients of PLSR obtained during stability investigation S10

Supplemental Table S1: Laboratory strains used in the study and cultivation times S11

Supplemental Table S2A: Model predictions for individual strains/patient isolates for the training data set S12

Supplemental Table S2B: Model predictions for individual patient isolates for the unknown test data set S16

Supplemental Table S3: Summary on model performance of the 4 tested models S18

Supplemental Table S4: Overview of methods used to identify *S. pneumoniae* S19

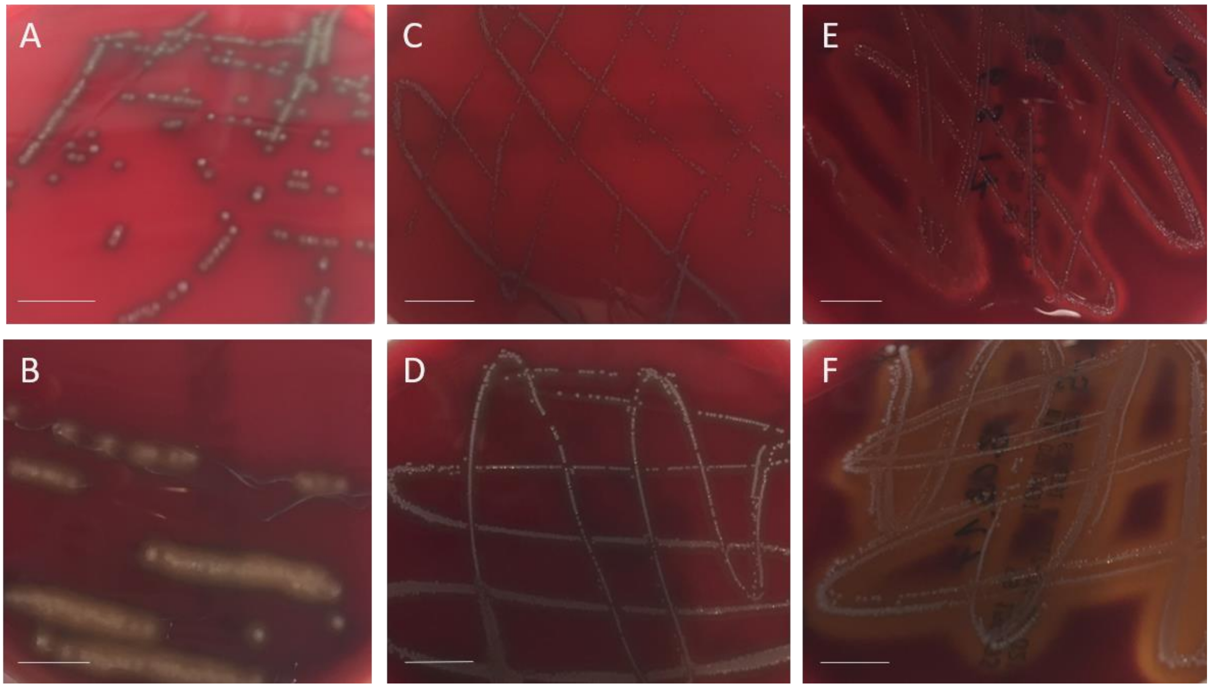

Supplemental Figure S1: Macroscopic characteristics of some *Streptococcus* species on blood agar plates after different cultivation times: 24 h top row (**A, C, E)** and three days bottom row (**B, D, F)**. **A, B**: *S. pneumoniae* ATCC49619 showing alpha-hemolysis. The colonies of *S. pneumoniae* ATCC49619 developed a central dent in the middle of the colonies after three days (B). This circumstance was probably due to autolysis behavior, whereby the bacteria died in the middle of the colonies (42). **C, D:** *S. sanguinis* DSMZ20567 showing alpha-hemolysis. **E/F:** *S. dysgalactiae* DSMZ6176 showing beta-hemolysis. Scale bar is 1 cm.

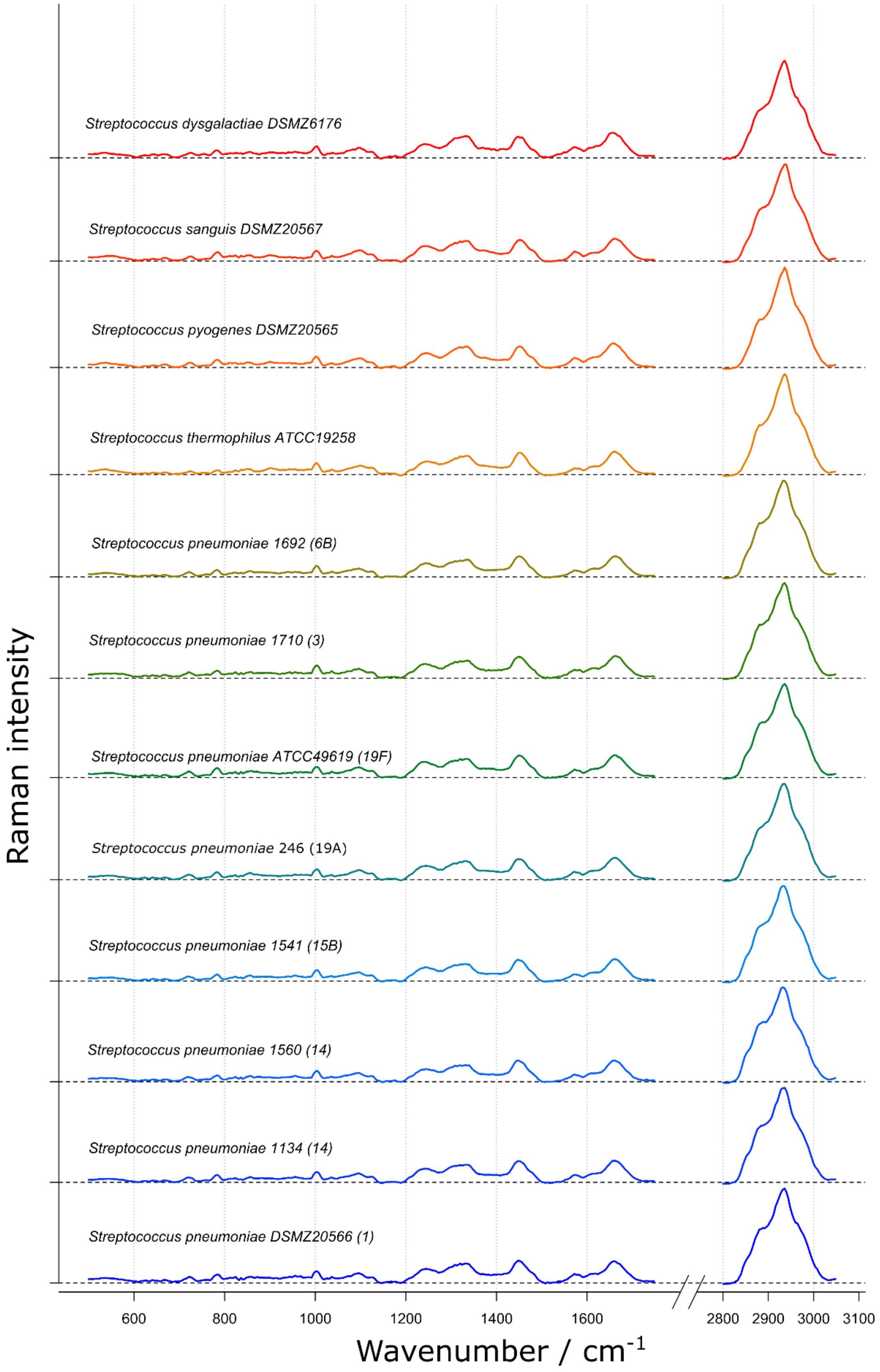

Supplemental Figure S2: Mean spectra of laboratory strains. From bottom to top: *S. pneumoniae* DSMZ 20566 (serotype 1), *S. pneumoniae* 1134 (serotype 14), *S. pneumoniae* 1560 (serotype 14), *S. pneumoniae* 1541 (serotype 15B), *S. pneumoniae* 246 (serotype 19A), *S. pneumoniae* ATCC49619 (serotype 19F), *S. pneumoniae* 1710 (serotype 3), *S. pneumoniae* 1692 (serotype 6B), *S. thermophilus* ATCC19258, *S. pyogenes* DSMZ 20565, *S. sanguinis* DSMZ 20567, *S. dysgalactiae* DSMZ 6176.

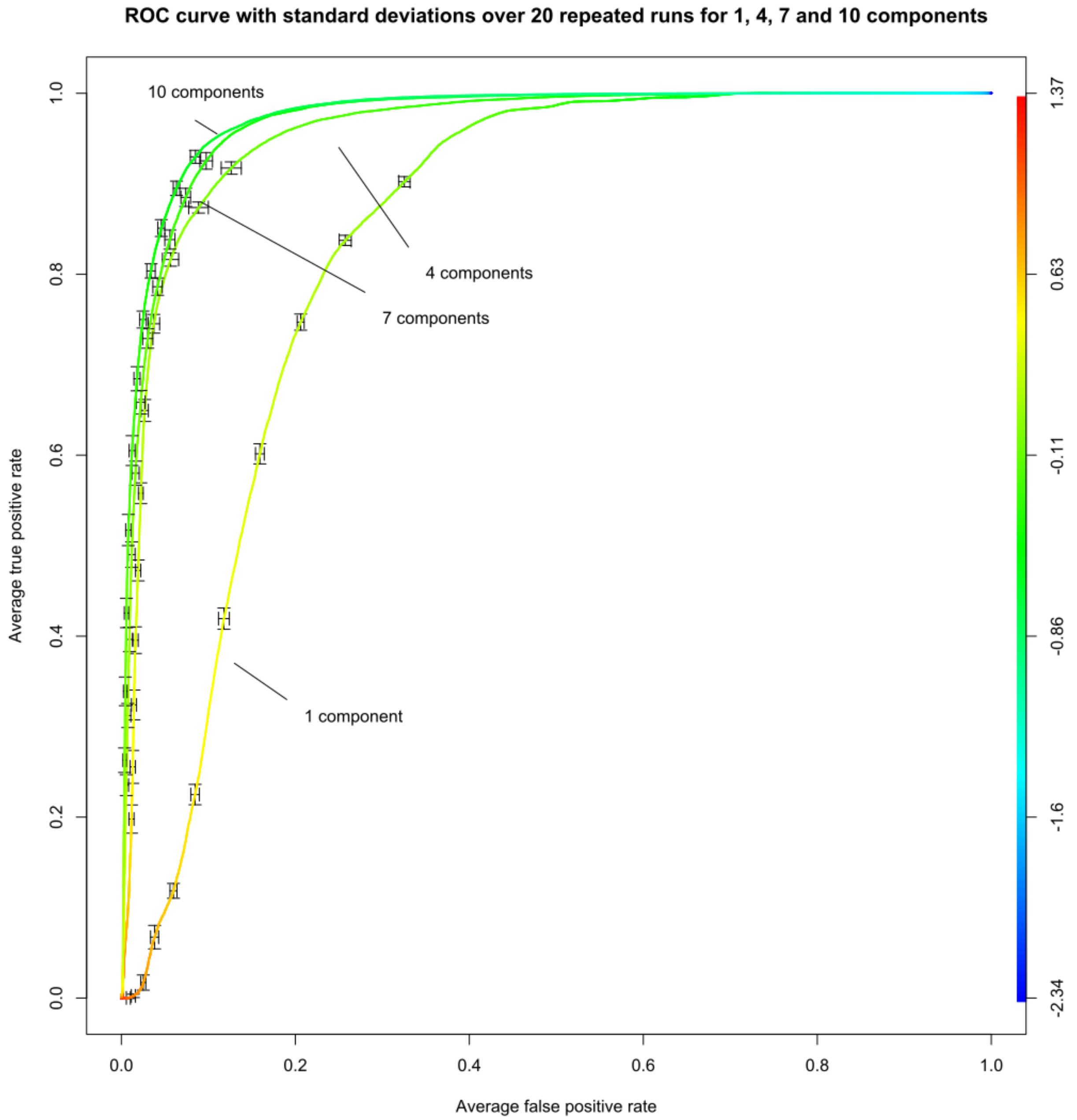

Supplemental Figure S3: **ROC curve for optimization of PLS-DA model based on vector normalized data**. ROC curve used for model optimization. The curves for models including 1, 4, 7 and 10 components are displayed exemplarily. The best model is chosen by smallest Euclidean distance to the upper left corner (1.0, 0.0) considering one standard deviation depicted as error bars to reduce model complexity and avoid overfitting.

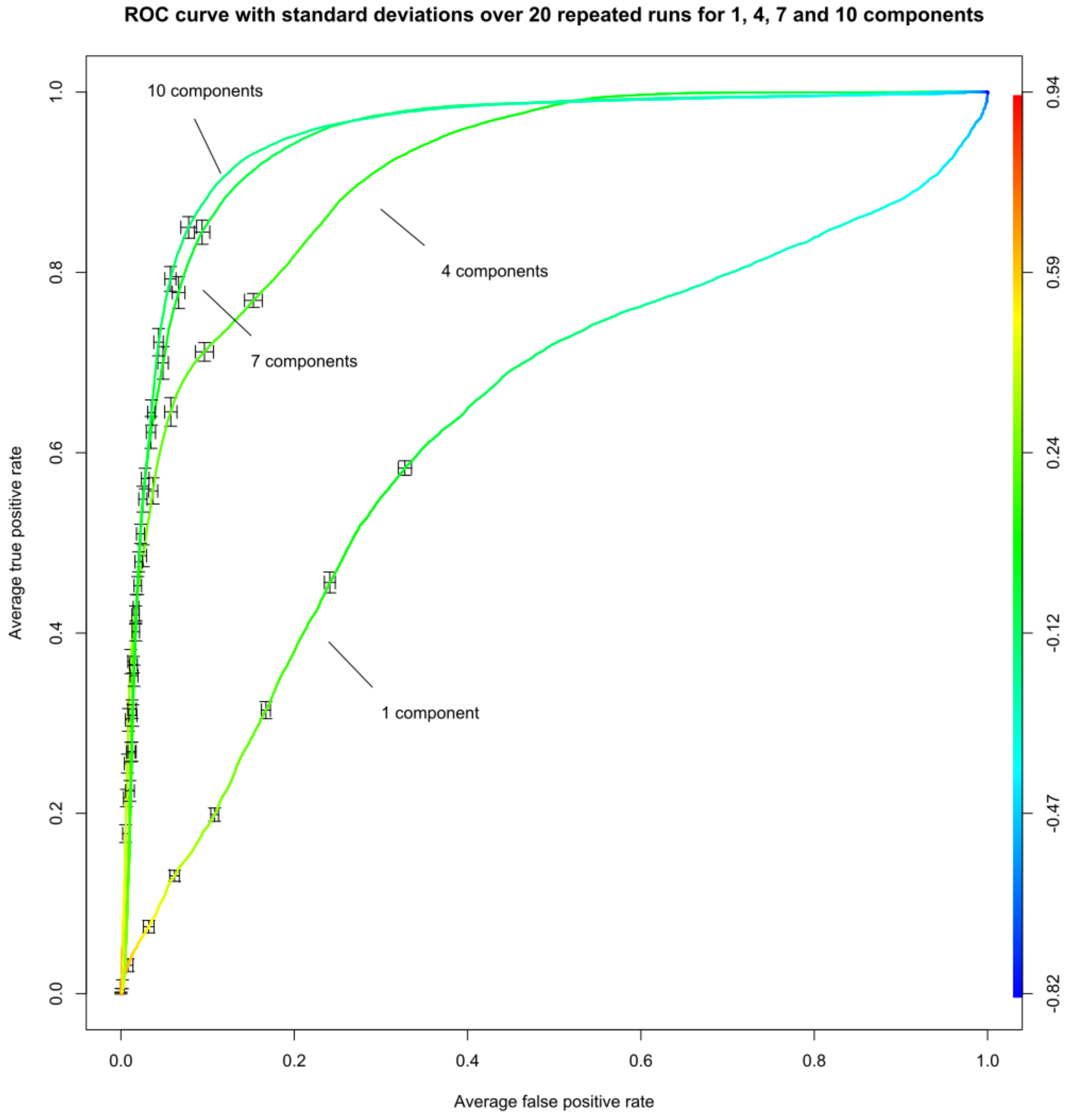

Supplemental Figure S4: **ROC curve for optimization of PLS-DA model based on data without vector normalization**. ROC curve used for model optimization. The curves for models including 1, 4, 7 and 10 components are displayed exemplarily. The best model is chosen by smallest Euclidean distance to the upper left corner (1.0, 0.0) considering one standard deviation depicted as error bars to reduce model complexity and avoid overfitting.

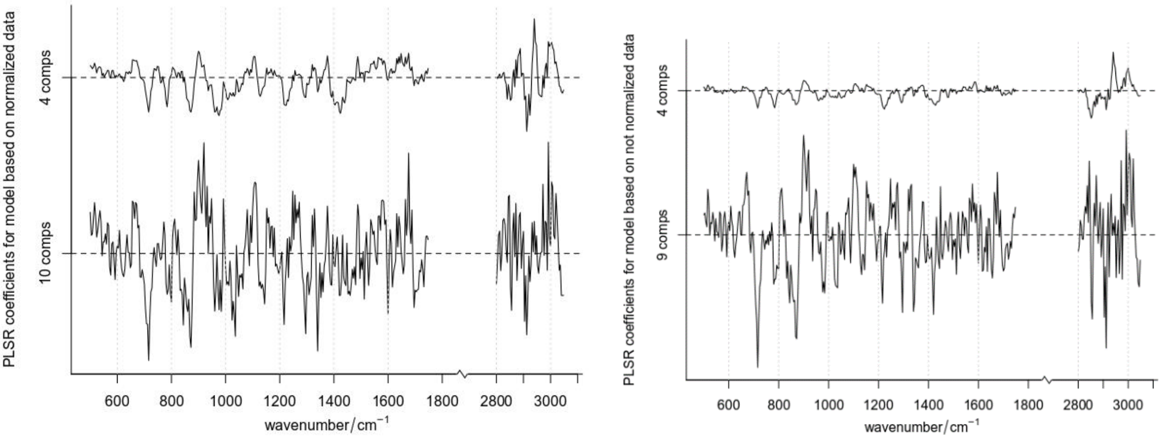

Supplemental Figure S5: PLS coefficients for models: Coefficients of the PLS regression are shown for the models using the optimal (bottom) and manually selected (top) number of components for models with (left) and without (right) vector normalization applied to the spectral data.

The PLSR loadings depicted in Figure 3, main manuscript, become quite noisy with 5 components, so using all components from optimization might carry the risk of overfitting the model. Therefore, it was decided to build two additional models using only 4 components. Note, that this selection is rather subjective to the authors' noise recognition behavior and was additionally guided by inspection of ROC curves for some of the 20 iterations during model optimization (not shown here, similar to Supplemental Figures S3 and S4).

The PLS regression coefficients plots above for the 4 models reveal, that using a lower number of components lead to much less noisy and thus, more reliable and interpretable coefficients likely to give better model performance due to less overfitting. Interestingly, the features of the regression coefficients show comparable peak heights across the whole spectrum. This shows that spectral information is used across the whole spectrum in a similar importance. Furthermore, using 4 components leads to similar coefficients for both types of preprocessing, although using normalization leads to slightly higher peak heights together with slightly higher noise levels.

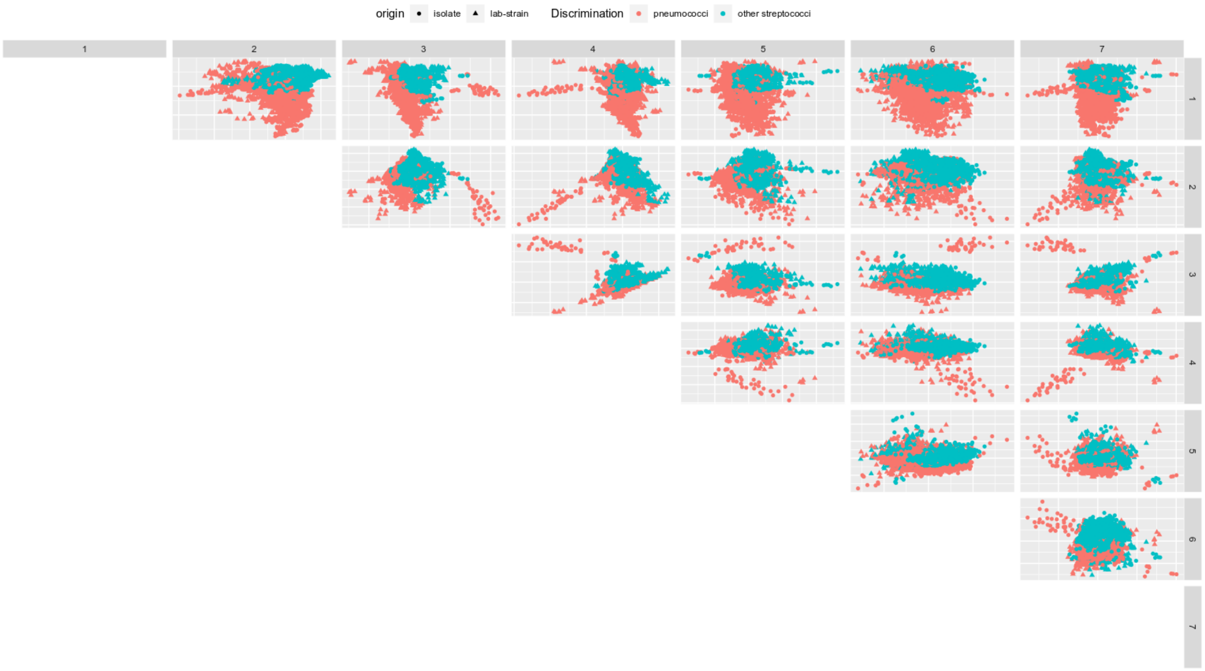

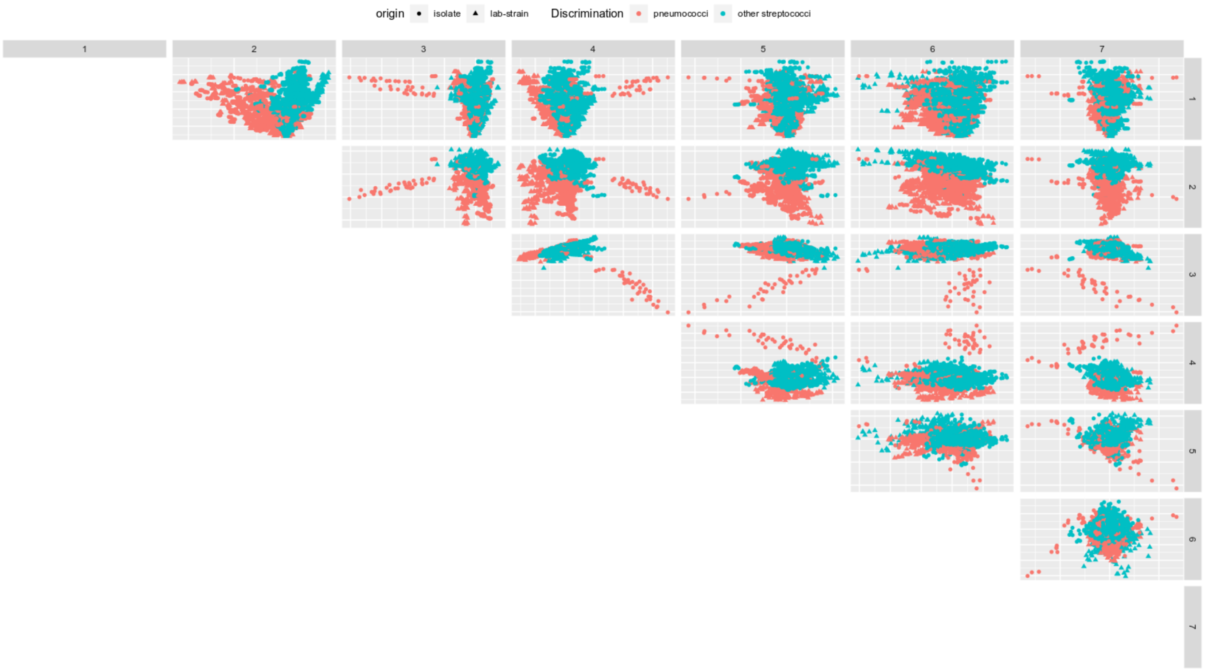

Supplemental Figure S6: **Binary PLSR model for the differentiation of *S. pneumoniae* and other streptococci:** Pairs plot of PLS scores for both models colored according classes (*S. pneumoniae* in red vs. other streptococci in turquoise). The score plot of the first two components (Comp1 and Comp 2) is shown in Figure 4, main manuscript. A different color coding of the same data is displayed in Supplemental Figure S5. Top shows data for model using normalized spectra, bottom data from spectra without normalization.

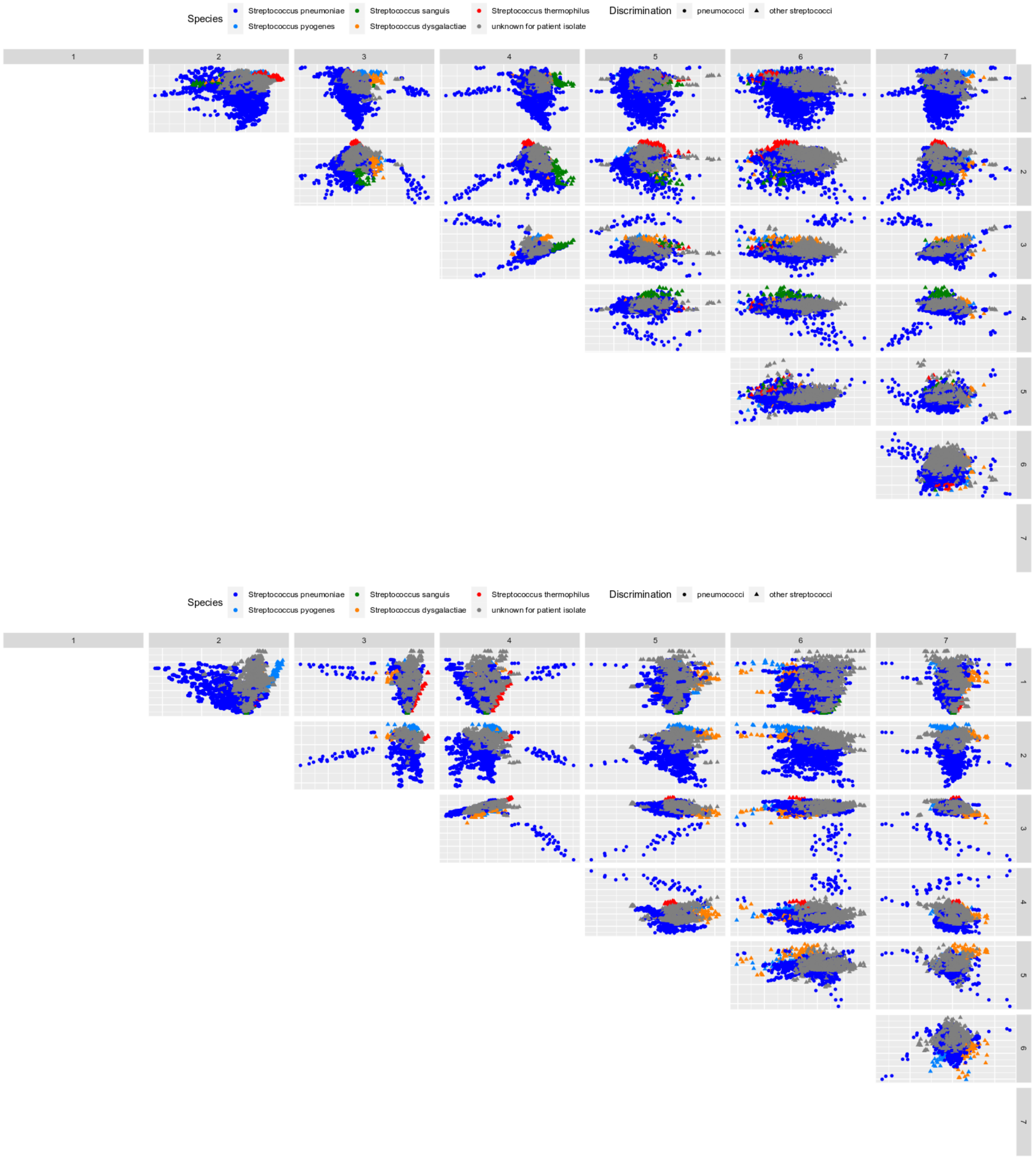

Supplemental Figure S7: **Binary PLSR model for the differentiation of *S. pneumoniae* and other streptococci:** Pairs plot of PLS scores for both models colored according to species (*S. pneumoniae,* dark blue, *S. pyogenes,* light blue, *S. sanguinis,* green, *S. dysgalactiae,* orange, *S. thermophilus,* red, viridians streptococci from patient isolates, grey). The score plot according to classes is shown in Supplemental Figure S4). Top shows data for model using normalized spectra, bottom data from spectra without normalization.

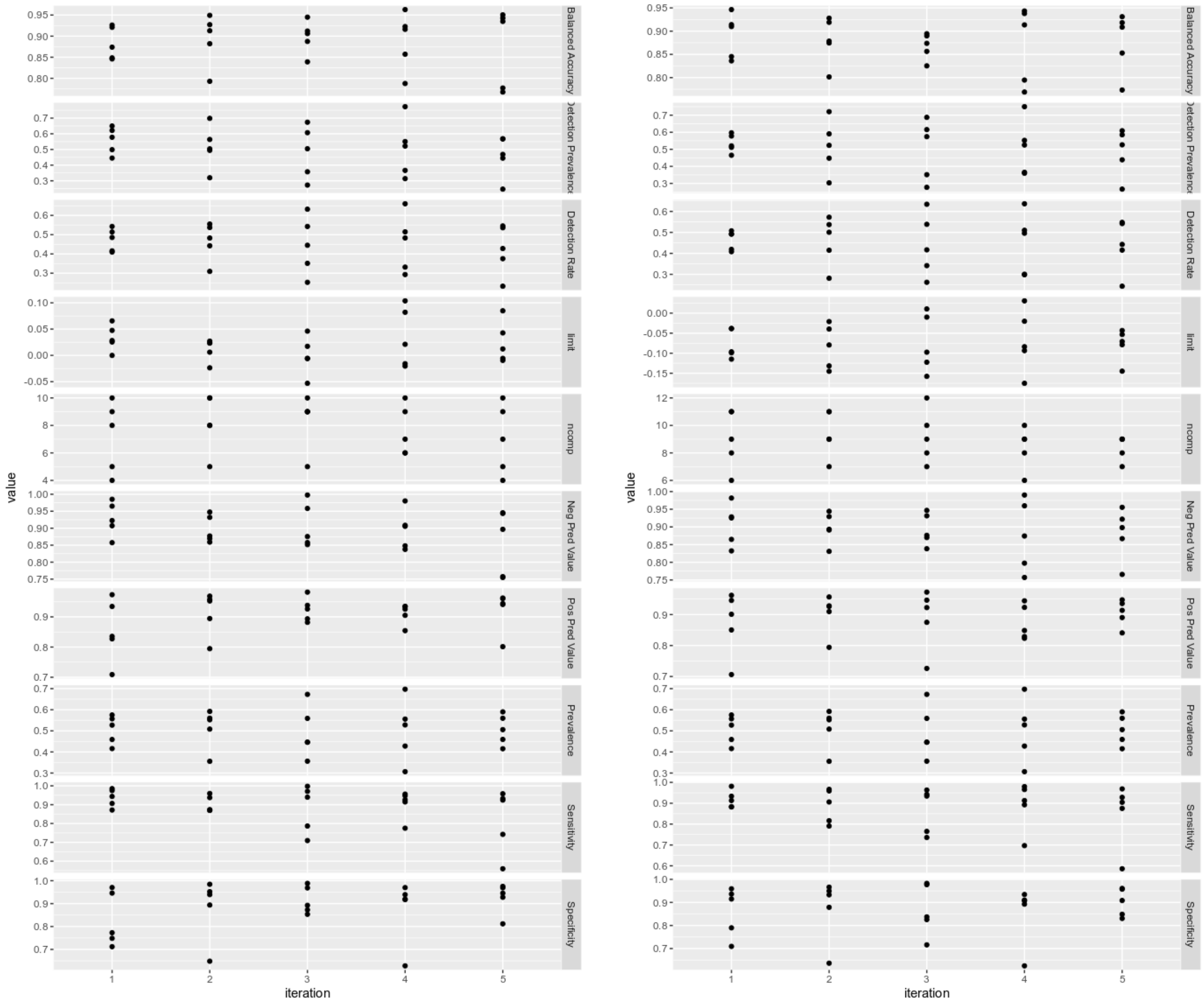

Supplemental Figure S8: **Model stability during outer cross-validation.** Performance for the model with (left) and without (right) vector normalization. Each dot represents the performance/value for a single fold, while remaining four folds were used for model optimization. The process is repeated 5 times depicted as iterations. A broad range of hyperparameters (ncomp for number of chosen components and limit for discrimination threshold) indicates instability of the optimization process.

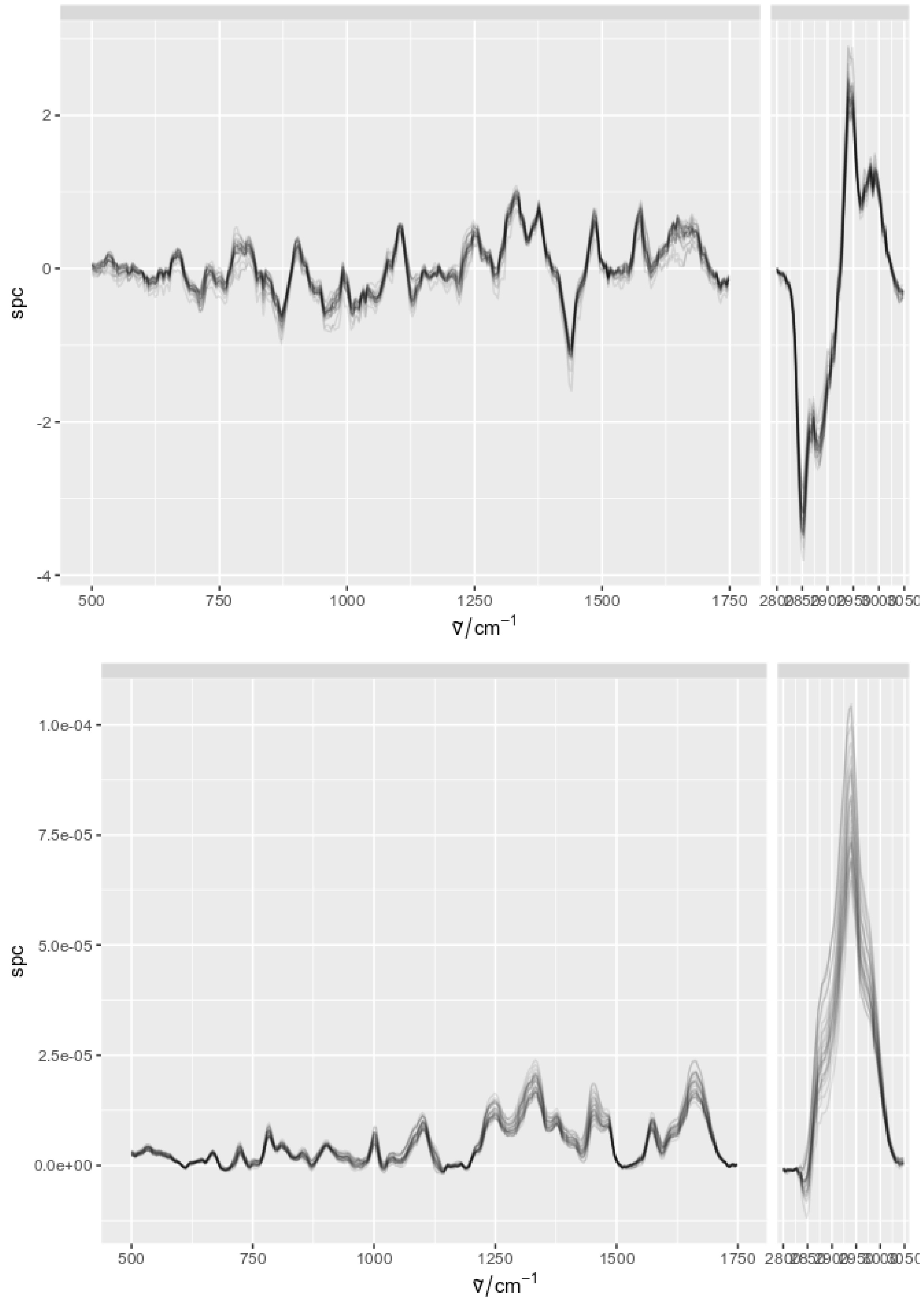

Supplemental Figure S9: **Regression coefficients of PLSR obtained during stability investigation**. Regression coefficients obtained during 5-times repeated model optimization based on data with (top) and without (bottom) vector normalization.

Supplemental Table S1A: Laboratory strains used in the study and cultivation times.

Species	Strain	Serotype	Replicate	Cultivation time
S. pneumoniae	*S. pneumoniae* ATCC49619	19F	2, 3, 5	20-24 h
			4	3 d
	S. pneumoniae DSMZ20566	1	2, 3, 5	20-24 h
			4	3 d
	S. pneumoniae 246	19A	1, 2, 3	20-24 h
	S. pneumoniae 1134	14	1, 2, 3	20-24 h
	S. pneumoniae 1541	15B	1, 2, 3	20-24 h
	S. pneumoniae 1560	14	1, 2, 3	20-24 h
	S. pneumoniae 1692	6B	1, 2, 3	20-24 h
	S. pneumoniae 1710	3	1, 2, 3	20-24 h
S. dysgalactiae	*S. dysgalactiae* DSMZ6176		2, 3, 5	20-24 h
			4	3 d
S. thermophilus	*S. thermophilus* ATCC19258		2, 3, 5	20-24 h
			4	3 d
S. sanguinis	*S. sanguinis* DSMZ20567		2, 3, 5	20-24 h
			4	3 d
S. pyogenes	*S. pyogenes* DSMZ20565		2, 3, 5	20-24 h
			4	3d

Supplemental Table S1B: Overview of clinical isolates. (note, this table contains information of training and test data set. For more information see Table S2)

Bacterial class	Patient material	# of patients	Total spectra
S. pneumoniae	blood culture	6	2950
S. pneumoniae	bronchoalveolar lavage	7	3179
S. pneumoniae	ear swab	1	450
S. pneumoniae	eye swab	3	1535
S. pneumoniae	nose lavage	3	1050
S. pneumoniae	nose swab	2	850
S. pneumoniae	nose throat swab	1	500
S. pneumoniae	sputum	2	750
S. pneumoniae	throat swab	1	443
S. pneumoniae	tracheal broncheal secretion	1	450
S. pneumoniae	wound swab	1	525
other streptococci	bronchoalveolar lavage	10	4900
other streptococci	eye swab	1	500
other streptococci	mouth swab	2	950
other streptococci	nose lavage	1	489
other streptococci	nose throat swab	9	4142
other streptococci	sputum	21	9317
other streptococci	throat lavage	4	1881
other streptococci	throat swab	9	4325
other streptococci	tracheal broncheal secretion	2	950

Supplemental Table S2A: Model predictions for individual strains/patient isolates for the training data set

Isolate	Origin	Microbiological assignment	norm.10	norm.4	no-norm.9	no-norm.4
S. thermophilus ATCC19258	lab strain	other streptococci	other streptococci (0/200)	other streptococci (0/200)	pneumococci (165/35)	pneumococci (165/35)
S. pneumoniae ATCC49619	lab strain	pneumococci	other streptococci (88/104)	other streptococci (92/100)	pneumococci (177/15)	pneumococci (177/15)
S. pyogenes DSMZ20565	lab strain	other streptococci	other streptococci (0/192)	other streptococci (0/192)	other streptococci (35/157)	other streptococci (35/157)
S. pneumoniae 1134	lab strain	pneumococci	pneumococci (121/29)	pneumococci (130/20)	other streptococci (40/110)	other streptococci (40/110)
S. pneumoniae 1541	lab strain	pneumococci	pneumococci (118/32)	pneumococci (125/25)	other streptococci (50/100)	other streptococci (50/100)
S. pneumoniae 1560	lab strain	pneumococci	pneumococci (123/22)	pneumococci (130/15)	pneumococci (95/50)	pneumococci (95/50)
S. pneumoniae 1692	lab strain	pneumococci	pneumococci (52/48)	pneumococci (54/46)	pneumococci (60/40)	pneumococci (60/40)
S. pneumoniae 1710	lab strain	pneumococci	pneumococci (52/38)	pneumococci (55/35)	pneumococci (85/5)	pneumococci (85/5)
S. pneumoniae 249	lab strain	pneumococci	pneumococci (78/72)	pneumococci (91/59)	pneumococci (126/24)	pneumococci (127/23)
S. pneumoniae DSMZ20566	lab strain	pneumococci	pneumococci (119/81)	pneumococci (126/74)	pneumococci (200/0)	pneumococci (200/0)
S. sanguinis DSMZ20567	lab strain	other streptococci	other streptococci (0/194)	other streptococci (0/194)	pneumococci (109/85)	pneumococci (109/85)
S. dysgalactiae DSMZ6176	lab strain	other streptococci	other streptococci (0/96)	other streptococci (0/96)	other streptococci (5/91)	other streptococci (5/91)
Patient_001	bronchoalveolar lavage	pneumococci	other streptococci (0/50)	other streptococci (6/44)	pneumococci (35/15)	pneumococci (35/15)
Patient_002	nose lavage	other streptococci	other streptococci (0/48)	other streptococci (0/48)	pneumococci (28/20)	pneumococci (28/20)
Patient_003	Throat lavage	other streptococci	pneumococci (24/23)	pneumococci (30/17)	other streptococci (17/30)	other streptococci (17/30)

Patient_004	eye swab	pneumococci	pneumococci (50/0)	pneumococci (50/0)	other streptococci (17/33)	other streptococci (17/33)
Patient_005	nose swab	pneumococci	pneumococci (50/0)	pneumococci (50/0)	other streptococci (0/50)	other streptococci (0/50)
Patient_006	blood culture	pneumococci	pneumococci (50/0)	pneumococci (50/0)	other streptococci (20/30)	other streptococci (20/30)
Patient_007	bronchoalveolar lavage	pneumococci	pneumococci (45/0)	pneumococci (45/0)	other streptococci (5/40)	other streptococci (5/40)
Patient_008	sputum	other streptococci	other streptococci (0/50)	other streptococci (0/50)	other streptococci (10/40)	other streptococci (10/40)
Patient_009	sputum	pneumococci	pneumococci (30/20)	pneumococci (36/14)	pneumococci (30/20)	pneumococci (30/20)
Patient_010	sputum	other streptococci	other streptococci (2/30)	other streptococci (4/28)	pneumococci (22/10)	pneumococci (22/10)
Patient_011	Throat lavage	other streptococci	other streptococci (0/45)	other streptococci (0/45)	pneumococci (25/20)	pneumococci (25/20)
Patient_012	sputum	other streptococci	other streptococci (0/44)	other streptococci (0/44)	other streptococci (19/25)	other streptococci (19/25)
Patient_013	bronchoalveolar lavage	other streptococci	other streptococci (0/50)	other streptococci (0/50)	other streptococci (5/45)	other streptococci (5/45)
Patient_014	blood culture	pneumococci	pneumococci (35/15)	pneumococci (36/14)	pneumococci (50/0)	pneumococci (50/0)
Patient_015	blood culture	pneumococci	pneumococci (50/0)	pneumococci (50/0)	other streptococci (20/30)	other streptococci (20/30)
Patient_016	blood culture	pneumococci	pneumococci (25/15)	pneumococci (25/15)	other streptococci (5/35)	other streptococci (5/35)
Patient_017	tracheal broncheal secretion	pneumococci	other streptococci (0/45)	other streptococci (5/40)	other streptococci (16/29)	other streptococci (16/29)
Patient_018	ear swab	pneumococci	other streptococci (5/40)	other streptococci (5/40)	other streptococci (5/40)	other streptococci (5/40)
Patient_019	sputum	pneumococci	other streptococci (5/20)	other streptococci (5/20)	pneumococci (25/0)	pneumococci (25/0)
Patient_020	blood culture	pneumococci	pneumococci (40/15)	pneumococci (41/14)	pneumococci (30/25)	pneumococci (30/25)
Patient_021	blood culture	pneumococci	pneumococci (50/0)	pneumococci (50/0)	pneumococci (40/10)	pneumococci (40/10)
Patient_022	wound swab	pneumococci	pneumococci (32/20)	pneumococci (37/15)	other streptococci (23/29)	other streptococci (24/28)
Patient_023	bronchoalveolar lavage	pneumococci	pneumococci (50/0)	pneumococci (50/0)	other streptococci (15/35)	other streptococci (15/35)
Patient_024	throat swab	pneumococci	pneumococci (33/9)	pneumococci (36/6)	pneumococci (42/0)	pneumococci (42/0)
Patient_025	sputum	other streptococci	other streptococci (9/36)	other streptococci (14/31)	other streptococci (15/30)	other streptococci (15/30)
Patient_026	mouth swab	other streptococci	pneumococci (35/10)	pneumococci (36/9)	other streptococci (5/40)	other streptococci (5/40)
Patient_027	bronchoalveolar lavage	other streptococci	other streptococci (14/36)	pneumococci (30/20)	other streptococci (20/30)	other streptococci (20/30)
Patient_028	sputum	other streptococci	other streptococci (22/28)	pneumococci (28/22)	other streptococci (15/35)	other streptococci (15/35)

Patient_029	sputum	other streptococci	other streptococci (0/45)	other streptococci (0/45)	other streptococci (5/40)	other streptococci (5/40)
Patient_030	throat swab	other streptococci	pneumococci (23/12)	pneumococci (26/9)	pneumococci (35/0)	pneumococci (35/0)
Patient_031	nose throat swab	other streptococci	other streptococci (0/54)	other streptococci (0/54)	other streptococci (0/54)	other streptococci (0/54)
Patient_032	nose throat swab	other streptococci	other streptococci (0/50)	other streptococci (0/50)	other streptococci (0/50)	other streptococci (0/50)
Patient_033	throat swab	other streptococci	other streptococci (0/50)	other streptococci (0/50)	other streptococci (0/50)	other streptococci (0/50)
Patient_034	Throat lavage	other streptococci	other streptococci (16/28)	other streptococci (16/28)	pneumococci (24/20)	pneumococci (24/20)
Patient_035	throat swab	other streptococci	other streptococci (0/50)	other streptococci (0/50)	other streptococci (5/45)	other streptococci (5/45)
Patient_036	nose lavage	pneumococci	pneumococci (50/0)	pneumococci (50/0)	pneumococci (35/15)	pneumococci (35/15)
Patient_037	nose swab	pneumococci	pneumococci (32/3)	pneumococci (35/0)	other streptococci (0/35)	other streptococci (0/35)
Patient_038	sputum	other streptococci	other streptococci (0/45)	other streptococci (1/44)	other streptococci (0/45)	other streptococci (0/45)
Patient_039	bronchoalveolar lavage	other streptococci	other streptococci (0/45)	other streptococci (0/45)	other streptococci (0/45)	other streptococci (0/45)
Patient_040	bronchoalveolar lavage	other streptococci	other streptococci (0/50)	other streptococci (0/50)	other streptococci (5/45)	other streptococci (5/45)
Patient_041	bronchoalveolar lavage	other streptococci	other streptococci (0/50)	other streptococci (0/50)	other streptococci (10/40)	other streptococci (10/40)
Patient_042	bronchoalveolar lavage	other streptococci	other streptococci (0/45)	other streptococci (0/45)	other streptococci (0/45)	other streptococci (0/45)
Patient_043	sputum	other streptococci	other streptococci (0/50)	other streptococci (0/50)	other streptococci (0/50)	other streptococci (0/50)
Patient_044	throat swab	other streptococci	other streptococci (0/50)	other streptococci (0/50)	other streptococci (10/40)	other streptococci (10/40)
Patient_045	bronchoalveolar lavage	pneumococci	pneumococci (45/5)	pneumococci (45/5)	other streptococci (0/50)	other streptococci (0/50)
Patient_046	nose throat swab	pneumococci	pneumococci (50/0)	pneumococci (50/0)	other streptococci (5/45)	other streptococci (5/45)
Patient_047	eye swab	pneumococci	pneumococci (50/3)	pneumococci (50/3)	other streptococci (5/48)	other streptococci (5/48)
Patient_048	sputum	other streptococci	other streptococci (4/46)	other streptococci (6/44)	other streptococci (10/40)	other streptococci (10/40)
Patient_049	nose throat swab	other streptococci	other streptococci (0/50)	other streptococci (0/50)	other streptococci (0/50)	other streptococci (0/50)
Patient_050	nose lavage	pneumococci	pneumococci (50/0)	pneumococci (50/0)	pneumococci (45/5)	pneumococci (45/5)
Patient_051	sputum	other streptococci	other streptococci (0/40)	other streptococci (0/40)	other streptococci (5/35)	other streptococci (5/35)
Patient_052	bronchoalveolar lavage	pneumococci	other streptococci (19/31)	pneumococci (25/25)	pneumococci (35/15)	pneumococci (35/15)
Patient_053	mouth swab	other streptococci	other streptococci (13/37)	pneumococci (28/22)	other streptococci (10/40)	other streptococci (10/40)

Patient_054	throat swab	other streptococci	other streptococci (0/50)	other streptococci (0/50)	other streptococci (5/45)	other streptococci (5/45)
Patient_055	bronchoalveolar lavage	other streptococci	other streptococci (0/50)	other streptococci (0/50)	other streptococci (0/50)	other streptococci (0/50)
Patient_056	nose throat swab	other streptococci	other streptococci (5/38)	other streptococci (14/29)	pneumococci (28/15)	pneumococci (28/15)
Patient_057	nose throat swab	other streptococci	other streptococci (9/29)	other streptococci (14/24)	pneumococci (33/5)	pneumococci (33/5)
Patient_058	nose throat swab	other streptococci	pneumococci (20/15)	pneumococci (20/15)	other streptococci (15/20)	other streptococci (15/20)
Patient_059	nose lavage	pneumococci	other streptococci (0/5)	other streptococci (0/5)	pneumococci (5/0)	pneumococci (5/0)

Supplemental Table S2B: Model predictions for individual patient isolates for the unknown test data set

Isolate	Origin	Microbiological assignment	norm.10	norm.4	no-norm.9	no-norm.4
Patient_060	sputum	other streptococci	pneumococci (9/0)	pneumococci (9/0)	pneumococci (9/0)	pneumococci (9/0)
Patient_061	sputum	other streptococci	pneumococci (20/15)	pneumococci (24/11)	other streptococci (10/25)	other streptococci (10/25)
Patient_062	sputum	other streptococci	pneumococci (25/14)	pneumococci (31/8)	pneumococci (29/10)	pneumococci (29/10)
Patient_063	sputum	other streptococci	other streptococci (4/35)	other streptococci (5/34)	pneumococci (20/19)	pneumococci (20/19)
Patient_064	sputum	other streptococci	other streptococci (4/46)	other streptococci (5/45)	other streptococci (10/40)	other streptococci (10/40)
Patient_065	sputum	other streptococci	other streptococci (7/41)	other streptococci (9/39)	pneumococci (25/23)	pneumococci (25/23)
Patient_066	sputum	other streptococci	other streptococci (3/47)	other streptococci (10/40)	pneumococci (30/20)	pneumococci (30/20)
Patient_067	eye swab	pneumococci	pneumococci (50/0)	pneumococci (50/0)	other streptococci (5/45)	other streptococci (5/45)
Patient_068	eye swab	other streptococci	other streptococci (4/46)	other streptococci (18/32)	pneumococci (25/25)	pneumococci (25/25)
Patient_069	throat swab	other streptococci	pneumococci (45/5)	pneumococci (49/1)	other streptococci (15/35)	other streptococci (15/35)
Patient_070	sputum	other streptococci	other streptococci (6/44)	other streptococci (15/35)	other streptococci (0/50)	other streptococci (0/50)
Patient_071	nose throat swab	other streptococci	other streptococci (17/33)	pneumococci (30/20)	other streptococci (0/50)	other streptococci (0/50)
Patient_072	bronchoalveolar lavage	pneumococci	other streptococci (14/31)	other streptococci (19/26)	pneumococci (45/0)	pneumococci (45/0)
Patient_073	nose throat swab	other streptococci	other streptococci (0/43)	other streptococci (0/43)	other streptococci (13/30)	other streptococci (13/30)
Patient_074	nose throat swab	other streptococci	other streptococci (6/44)	other streptococci (8/42)	other streptococci (0/50)	other streptococci (0/50)
Patient_075	throat swab	other streptococci	pneumococci (34/11)	pneumococci (38/7)	other streptococci (5/40)	other streptococci (5/40)
Patient_076	bronchoalveolar lavage	pneumococci	other streptococci (5/22)	other streptococci (6/21)	pneumococci (27/0)	pneumococci (27/0)
Patient_077	throat swab	other streptococci	other streptococci (0/50)	other streptococci (0/50)	other streptococci (0/50)	other streptococci (0/50)
Patient_078	Throat lavage	other streptococci	other streptococci (1/49)	other streptococci (7/43)	other streptococci (0/50)	other streptococci (0/50)
Patient_079	sputum	other streptococci	other streptococci (0/54)	other streptococci (0/54)	other streptococci (10/44)	other streptococci (10/44)

Patient_080	sputum	other streptococci	pneumococci (50/0)	pneumococci (50/0)	other streptococci (15/35)	other streptococci (15/35)
Patient_081	tracheal broncheal secretion	other streptococci	other streptococci (0/50)	other streptococci (4/46)	other streptococci (5/45)	other streptococci (5/45)
Patient_082	bronchoalveolar lavage	other streptococci	other streptococci (0/50)	other streptococci (0/50)	other streptococci (15/35)	other streptococci (15/35)
Patient_083	sputum	other streptococci	other streptococci (0/50)	other streptococci (0/50)	other streptococci (5/45)	other streptococci (5/45)
Patient_084	bronchoalveolar lavage	other streptococci	pneumococci (45/5)	pneumococci (50/0)	other streptococci (10/40)	other streptococci (10/40)
Patient_085	bronchoalveolar lavage	other streptococci	other streptococci (0/50)	other streptococci (0/50)	other streptococci (5/45)	other streptococci (5/45)
Patient_086	tracheal broncheal secretion	other streptococci	other streptococci (3/42)	other streptococci (5/40)	other streptococci (20/25)	other streptococci (20/25)
Patient_087	throat swab	other streptococci	other streptococci (0/50)	other streptococci (0/50)	other streptococci (0/50)	other streptococci (0/50)

Supplemental Table S3: Summary on model performance of the 4 tested models

A: Summary on model performance of the 4 tested models during auto-prediction (training data set). The positive class is pneumococci. The number in brackets represents the corresponding value after majority vote per patient.

Prepro-cessing	Number of compo-nents	Discrimination threshold	Sensitivity	Specificity	Positive predictive value	Balanced accuracy
Vector normali-zation	10	-0.01	68.9 (78.8)	91.3 (89.5)	89.1 (86.7)	80.1 (84.1)
	4	0.043	72.7 (81.8)	88.2 (81.6)	86.3 (79.4)	80.4 (81.7)
No normali-zation	9	-0.091	57.8 (51.5)	69.2 (76.3)	65.9 (65.4)	63.5 (63.9)
	4	-0.090	57.9 (51.5)	69.2 (76.3)	65.9 (65.4)	63.6 (63.9)

B: Summary on model performance of the 4 tested models during prediction of test data. The positive class is pneumococci. The number in brackets represents the corresponding value after majority vote per patient.

Preprocessing	Number of components	Discrimination threshold	Sensitivity	Specificity	Positive predictive value	Balanced accuracy
Vector normalization	10	-0.01	56.6 (33.3)	75.5 (72.0)	19.6 (12.5)	66.0 (52.7)
	4	0.043	61.5 (33.3)	68.3 (68.0)	17.0 (11.1)	64.9 (50.7)
No normalization	9	-0.091	63.1 (66.7)	76.1 (76.0)	21.8 (25.0)	69.6 (71.3)
	4	-0.090	63.1 (66.7)	76.1 (76.0)	21.8 (25.0)	69.6 (71.3)

It can be seen, that using only 4 components shifts the discrimination threshold to higher values leading to higher sensitivity for both preprocessings, but overall accuracy remains nearly the same. This indicates, that a lower number of components is sufficient for discrimination of *S. pneumoniae* and other streptococci, as was already seen in the scores plots.

Supplemental Table S4: Overview of methods used in clinical routine diagnostics to identify *S. pneumoniae*. Please note, serotyping was only done for *S. pneumoniae* from invasive infection (blood culture).

Patient ID	Species (ST)	patient material	cultivation / genetic	optochin sensitivity	Maldi-TOF-MS	Serotyping
001	*Streptococcus pneumoniae*	bronchoalveolar lavage	further processing /isolation	+	+	
004	*Streptococcus pneumoniae*	eye swab	+	+		
005	*Streptococcus pneumoniae*	nose swab	+	+		
006	*Streptococcus pneumoniae* (22F)	blood culture	+			22F
007	*Streptococcus pneumoniae*	bronchoalveolar lavage	+	+		
009	*Streptococcus pneumoniae*	sputum	+	+		
014	*Streptococcus pneumoniae* (15C)	blood culture	+		+	15C
015	*Streptococcus pneumoniae* (11A)	blood culture	further processing /isolation		+	11A
016	*Streptococcus pneumoniae*	blood culture	+			NA
017	*Streptococcus pneumoniae*	tracheal bronchial secretion	+	+		
018	*Streptococcus pneumoniae*	ear swab	+	+	+	
019	*Streptococcus pneumoniae*	sputum	+	+		
020	*Streptococcus pneumoniae* (16F)	blood culture	+			
021	*Streptococcus pneumoniae* (23A)	blood culture	further processing /isolation	+	+	23A
022	*Streptococcus pneumoniae*	wound swab	+	+		
023	*Streptococcus pneumoniae*	bronchoalveolar lavage	+	+		
024	*Streptococcus pneumoniae*	throat swab	+			
036	*Streptococcus pneumoniae*	nose lavage	+	+		

037	*Streptococcus pneumoniae*	nose swab	+	+		
045	*Streptococcus pneumoniae*	bronchoalveolar lavage	+	+	+	
046	*Streptococcus pneumoniae*	nose throat swab		+	+	
047	*Streptococcus pneumoniae*	eye swab	further processing /isolation	+	+	
050	*Streptococcus pneumoniae*	nose lavage		+	+	
052	*Streptococcus pneumoniae*	bronchoalveolar lavage		+		
059	*Streptococcus pneumoniae*	nose lavage			+	
067	*Streptococcus pneumoniae*	eye swab		+		
072	*Streptococcus pneumoniae*	bronchoalveolar lavage	+			
076	*Streptococcus pneumoniae*	bronchoalveolar lavage	+			
